# Closing Editorial: Immunophenotyping in Autoimmune Diseases and Cancer 3.0

**DOI:** 10.3390/ijms25126311

**Published:** 2024-06-07

**Authors:** Gábor J. Szebeni, Attila Balog

**Affiliations:** 1 Laboratory of Functional Genomics, Core Facility, HUN-REN Biological Research Centre, Temesvári krt. 62, H6726 Szeged, Hungary; 2 Department of Internal Medicine, Hematology Centre, Faculty of Medicine, University of Szeged, H6725 Szeged, Hungary; 3 Department of Rheumatology and Immunology, Faculty of Medicine, Albert Szent-Gyorgyi Health Centre, University of Szeged, Kálvária sgt. 57, H6725 Szeged, Hungary

The mammalian immune system is a Janus-faced network of well-coordinated highly specialized cells and biomolecules. The sensitive balance of reactive and suppressive signals maintains homeostasis in a physiological state. However, under pathological conditions, this balance can be disrupted, leading to either excessive responsiveness as seen in autoimmune diseases, or suppression, as seen in cancer. In severe autoimmunity, the overwhelming immune response leads to devastating diseases, such as rheumatoid arthritis, systemic lupus erythematosus, systemic sclerosis, and multiple sclerosis. Conversely, in cancer, immune cells, such as professional antigen-presenting cells and cytotoxic T-cells, fail to execute their physiological functions. Immunophenotyping using fluorochrome- or metal-labeled antibodies has become a widely used technology to reveal the functional heterogeneity of leukocytes and to monitor protein expression detected by single-cell flow cytometry [1]. Recent progress in fluorescence flow cytometry and mass cytometry has enabled high-dimensional resolution of the complex immunophenotype in both autoimmune diseases and cancer [2,3,4]. Therefore, we invited authors to publish their latest achievements related to the perturbation of the regulation of immune activation and the discovery of rare subpopulations of innate and adaptive immune players in autoimmune diseases or cancer. This Special Issue about immunophenotyping features works that describe different cellular subtypes and the discovery of key molecular factors and biomarkers in cancer or in autoimmune diseases.

Faragó et al. (contribution 1) applied fluorescently labeled lectins as a tool for immunophenotyping of the CT26 murine colorectal carcinoma model. The binding of six lectins to white blood cells, such as galectin-1 (Gal1), siglec-1 (Sig1), *Sambucus nigra* lectin (SNA), *Aleuria aurantia* lectin (AAL), *Phytolacca americana* lectin (PWM), and galectin-3 (Gal3), was assayed. Flow cytometric analysis of the splenocytes revealed increased binding of SNA, and AAL to CD3^+^ T-cells and CD11b^+^ myeloid cells; and increased siglec-1 and AAL binding to CD19^+^ B-cells in the tumor-bearing mice.

Liu J. et al. (contribution 2) demonstrated different expression patterns of the G protein-coupled estrogen receptor (GPER1) in esophageal squamous cell carcinoma (ESCC) and esophageal adenocarcinoma (EAC). The GPER1 is involved in several sex-related cancers; however, its expression level in esophageal carcinoma has been poorly investigated, and its role is not precisely defined, varying with histological types. The protein expression levels of GPER1 were detected by immunohistochemistry in a tissue microarray of EAC and ESCC. The average staining scores of GPER1 protein in the tissue microarray of EAC were significantly higher than those of normal esophageal samples, and the rate of positive staining increased with the grade of poor tumor differentiation. The scores of GPER1 protein in ESCC tissues were lower than those in normal tissues. The results of this study indicated that the expression levels of GPER1 are higher in EAC than in ESCC, which might be correlated with the dimorphic estrogen signaling pathway in different types of esophageal carcinoma.

Lu et al. (contribution 3) evaluated the association between Ferredoxin 1 (FDX1) expression and the prognostic survival of tumor patients and predicted the efficacy of immunotherapy response to antitumor drug sensitivity. FDX1 demonstrated correlations with immune and molecular subtypes, and displayed relationships with the tumor mutational burden (TMB), microsatellite instability (MSI), and DNA methylation within the tumor microenvironment. FDX1 exhibited a strong connection with immune checkpoint genes in the co-expression network. Elevated FDX1 expression has been linked to the enhanced effectiveness of PD-L1 blockade immunotherapy in melanoma, as observed in the GSE22155 and GSE172320 cohorts. Collectively, these findings propose that FDX1 could serve as a novel and valuable biomarker and represents an immunotherapeutic for augmenting immune responses in various human cancers when used in combination with immune checkpoint inhibitors.

Murthy et al. (contribution 4) investigated the relationship between expression of prostaglandin E synthase (PTGES) isoforms and the pathogenesis and regulation of pancreatic cancer. Their analysis identified higher expression of PTGES in pancreatic tumors compared to normal pancreatic tissues, suggesting an oncogenic function. Only PTGES1 expression was significantly correlated with worse prognosis of pancreatic cancer patients. Furthermore, utilizing cancer genome atlas data, PTGES was found to be positively correlated with epithelial–mesenchymal transition, metabolic pathways, mucin oncogenic proteins, and immune pathways in cancer cells. PTGES expression was also correlated with higher mutational burden in key driver genes such as TP53 and KRAS. Furthermore, their analysis indicated that the oncogenic pathway controlled by PTGES1 could be regulated via DNA methylation-dependent epigenetic mechanisms. Notably, the glycolysis pathway was positively correlated with PTGES and may fuel cancer cell growth. PTGES expression was also associated with downregulation of the MHC pathway and negatively correlated with CD8^+^ T-cell activation markers. In summary, their study established an association of PTGES expression with pancreatic cancer metabolism and the immune microenvironment.

Zhu et al. (contribution 5) reviewed circular RNAs (circRNAs), a class of endogenous long non-coding RNAs with a single-stranded circular structure. Most circRNAs are relatively stable, highly conserved, and specifically expressed in tissue during various cell and developmental stages. Many circRNAs have been discovered in oral squamous cell carcinoma (OSCC), one of the most severe and frequent forms of head and neck cancer today, characterized by poor prognosis and low overall survival rates. Due to its prevalence, OSCC is a global health concern, marked by genetic and epigenomic changes. However, the mechanism remains unclear. With advancements in biotechnology, a large number of circRNAs have been discovered in mammalian cells. In OSCC tissues, circRNAs are dysregulated and thus associated with the clinicopathological characteristics and prognosis of OSCC patients. Research studies have demonstrated that circRNAs can serve as biomarkers for OSCC diagnosis and treatment. Here, they summarized the properties, functions, and biogenesis of circRNAs, focusing on the progress of current research on circRNAs in OSCC.

Fraticelli et al. (contribution 6) conducted immunophenotypic analysis of Castleman disease (CD), a rare lymphoproliferative disorder including various clinicopathological subtypes. CD is divided into unicentric CD (UCD) and multicentric CD (MCD) based on its clinical course. MCD is further subdivided based on the etiological driver into herpes virus 8-related MCD (that can occur in the setting of HIV); MCD associated with POEMS syndrome (polyneuropathy, organomegaly, endocrinopathy, monoclonal protein, and skin changes); and idiopathic MCD (iMCD). The latter can also be divided into iMCD-TAFRO (thrombocytopenia, anasarca, fever, myelofibrosis, and organomegaly) and iMCD not otherwise specified. To date, CD pathogenesis is still uncertain, but CD may represent the histological and clinical result of heterogeneous pathomechanisms. On this basis, their study aimed to investigate the distribution of T-cell subsets in the clinicopathological spectrum of CD. They evaluated the CD4/CD8 ratio and the number of regulatory T (Treg) FOXP3^+^ cells in 28 CD cases. In total, 32% of cases showed a decreased CD4/CD8 ratio due to increased CD8^+^ T-cells, including cases of UCD, iMCD, and HHV8^+^ MCD. The Treg subset analysis revealed a significantly (*p* < 0.0001) lower mean number of FOXP3^+^ Treg cells in CD cases when compared with non-specific reactive lymph nodes. These findings may suggest that alterations in T-cell subpopulations that can lead to disruption of immune system control may contribute to the numerous changes in the different cellular compartments that characterize CD.

Hansen et al. (contribution 7) reviewed the involvement of lymphocytes in Graves’ disease (GD), a thyroid-specific autoimmune disease. In GD, the T-cell populations are markedly distinct, including increased levels of Th17 and follicular helper T (Tfh)-cells, while Treg cells appear to be impaired. Some B-cell subsets are autoreactive, and anti-TSHR (thyroid stimulating hormone receptor) autoantibodies are the key disease-causing outcome of this interplay. Though some consensus across phenotyping studies will be discussed here, there are also complexities that remain unresolved. A better understanding of the immunophenotype of Graves’ disease can lead to improved treatment strategies and novel drug targets.

Dai et al. (contribution 8) studied Crohn’s disease (CD), a highly heterogeneous inflammatory bowel disease characterized by a unique inflammatory phenotype of T-cells. They obtained single-cell expression profile data from 22 CDs or normal samples and performed cell annotation and cellular communication analysis. Through the intersection of T-cell marker genes, differential genes, and WGCNA (weighted gene co-expression network analysis) results, they identified T-cell specific key genes, their immune landscapes, and their potential pathogenesis, and validated these findings across multiple datasets and patient tissue samples. Molecular docking demonstrated that BIRC3 (baculoviral IAP repeat containing 3) and ANXA1 (Annexin A1) have strong binding properties to azathioprine and glucocorticoids in silico. Single-cell sequencing, targeting T-cell-related features in patients with Crohn’s disease, may aid in new diagnostic decisions, as well as the initial exploration of high-potential therapies.

Liu G. et al. (contribution 9) generated monoclonal antibodies against *Glaesserella parasuis* (*G. parasuis*), the etiological pathogen of Glässer’s disease, which causes significant economic losses in the pig industry. In this study, three monoclonal antibodies (mAbs)—5D11, 2H81, and 4F2- were generated against recombinant HbpA (rHbpA, heme-binding protein A) of *G. parasuis* SH0165 (serotype 5) by fusing SP2/0-Ag14 murine myeloma cells and spleen cells from BALB/c mice immunized with rHbpA. Indirect enzyme-linked immunosorbent assay (ELISA) and indirect immunofluorescence assay (IFA) demonstrated that the antibody designated as 5D11 showed a strong binding affinity with the HbpA protein and was chosen for subsequent experiments. Their results indicated that mAb 5D11 and EP-5D11 could potentially be used to develop serological diagnostic tools for *G. parasuis*.

**Future Perspectives**

Nearly a decade has passed since the introduction of mass cytometry offering high multiplex immunophenotyping up to 55 markers per sample, based on the detection of heavy metal tags [5]. Presently, spectral flow cytometry (SFC) has reached a breakthrough in multiplex fluorochrome-based immunophenotyping. Cutting-edge analyzers such as the BD FACSymphony™ A5 SE (up to 9 lasers with 50 detection channels), spectral single-cell sorters such as Cytek Aurora (up to 5 lasers with 64 channels), or the Thermo Fisher Scientific BigFoot (up to 9 lasers with 60 detection channels) have transformed the field [6]. The SFC offers effective discrimination of autofluorescence or background signals allowing for live cell analysis or cell sorting, unlike mass cytometry, where the cells are fixed and ionized during the analysis. Multiplex fluorescent flow cytometry may encounter issues with the deviation of emission spectra caused by the interaction of the fluorochromes/tandem dyes in multiplex panels. However, Sahir et al. optimized a 43-membered panel for the Cytek Aurora and demonstrated an investigation of 130 subpopulations of human PBMCs with single-cell resolution [7]. Nowadays, personalized medicine approach testing the patient-derived living cells with single-cell resolution may assist in decision-making for therapeutic intervention and ameliorate the prognosis of the patients. Recently, Pregrej et al. used a combination of two 22-color immunophenotyping panel while studying the single-cell drug responses of human PBMCs, derived from autoimmune patients [8]. With the right choice of antibody panels and relevant control samples, the bioinformatic analysis of single-cell data has become a cornerstone of high-multiplex immunophenotyping. Deciphering the single-cell immune landscape using unsupervised algorithms to reveal relevant differences with biological significance has an unequivocal importance in multiplex flow cytometry. The emergence of SFC required the development of new pipelines for the analysis of high-dimensional data generated by SFC; therefore, new workflows handling SFC data integrating previously published R-based packages have been published [9,10]. Spasic et al. developed advanced bioinformatic tools and published optimized SFC panels, 2 panels detecting 48 cell surface markers of PBMCs and a bone marrow panel consisting of 32 parameters for the analysis of hematological malignancies [11]. The concurrent advancement of flow cytometric technology and AI assisted data analysis has revolutionized single-cell immunophenotyping in the 21st century [12]. Challenges for future development of single-cell flow cytometry are the following: (a) the increase in the resolution of the detectors in conjunction with the improvement in the signal-to-noise ratio; (b) the application of higher numbers of detectors with narrow, 1–2 nm bandwidth; (c) the development of fluorescent dyes with narrow (5–10 nm) emission spectra; (d) almost real-time bioinformatic analysis for single-cell sorting from unsupervised clustering created metaclusters of particular cell populations.
